# The Infirmary Medical Superintendents' Society

**Published:** 1890-12-13

**Authors:** 


### The Infirmary Medical Superintendents' Society.

The monthly meeting of this society was held on Saturday, November 29th, at half-past three p.m., at the Lambeth Infirmary. Dr. Lloyd, the medical superintendent of the infirmary, showed the following interesting cases in the wards of the establishment.

Case 1. "Blenorrhagic Arthritis."—The patient was a man, 53 years of age, who stated that 16 years ago he was perfectly well. About this time he was admitted into Guy's Hospital for a rheumatism affection which first appeared in the feet and then in the knees. After he left the hospital, by the use of crutches and an invalid chair he was able to get about with difficulty. He had had a chronic gleet for several years. He was admitted into the infirmary six years ago, being completely helpless. He had a slight urethral stricture, and blindness following an attack of glaucoma. At the present time, he lay on his back helpless, the arms were semi-flexed at the elbows, the hands were placed in a prone position on the lower part of the abdomen. The wrists were strongly turned to the ulnar side, all the digits were flexed, the thumbs being extended. The skin of the hands and fingers presented a glossy appearance. The legs were extended, the toes semi-flexed. There was some skin eruption about the left knee-joint and the right leg. There was now considerable discolouration about the toes of the left foot. He was able to slightly move his thumbs, and could just raise the left elbow from the bed; no other movement of the limbs was possible. His pain was best relieved by four grain doses of carbonate of lithia. Case 2. A woman, aged 70, had been a cook, admitted with extreme "obesity" producing shortness of breadth. She had been treated on Towers-Smith's method. This consisted of a lean meat diet and hot-water. At first she had had four pounds of meat daily, together with six pints of hot water during the day, and one pint during the night. She also took a certain amount of walking exercise daily. At the end of about a week, her weight remaining stationary, the diet was reduced to two pounds of meat daily, and after this to one pound of meat and one pound of fish, the hot water being continued as before. She had no tea, sugar, or, in fact, anything else to eat or drink. Compound jalap powder was also occasionally given. Under this treatment her weight, which on October 23rd was 17 stone 5 lbs., was on November 27th 16 stone 11 lbs. Case 3 was that of a woman about 65 years of age. She had been brought in by the police, stated to have had a fit. When first seen there was no evidence of paralysis, her speech was good, but she had total loss of memory. For about a month after admission she walked about, but could give absolutely no account of herself. Suddenly one day she said her name was Bath; beyond this her mind was a perfect blank. At the present time she was unable to give a name to anything. When shown a watch she was unable to give it a name or to specify its use, the same was the case with a coin or pencil case. Case 4 was that of a woman with a large "prepatellar swelling." This had been tapped, and a small quantity of sanguineous fluid was drawn off. But the size of the tumour was not much decreased, and it appeared to be a multilocular cyst.

Dr. Lloyd also showed a case of skin disease which he believed to be a tertiary form of syphilis. The patient had been under his care on and off for the last fifteen years. The lesions were now confined to the internal angle of the right orbit and the upper eyelid, the extensor surface of the right forearm, and the upper regions of the back. Ten grain doses of iodide of potassium had failed to influence it. Some cases of tumours of the thyroid were also shown, together with a case of myxoedema. This last was a woman, who was shown before the Pathological Society in 1881. She presented much the same condition now as she did nearly ten years ago. There was no albumen. Since she was first brought under notice in 1881 she has had both scarlet fever and small-pox. With the former she was very ill, although her temperature was only 101, and she had intermittent albuminuria.

Dr. Blachford, the assistant medical officer, showed a case of tetanus occurring in a boy eleven years of age. He was admitted eleven days ago with the history of having received a blow on the head three days previously. On admission, the pulse was 120, and the temperature 101.2. He was sweating and in convulsions; his mouth was fixed and slightly drawn. The whole body was rigid, the abdomen exceedingly tense. The legs were quite stiff, the arms less so. He has fits at about intervals of twelve hours which appear to be of an epileptiform nature, in which he becomes livid and the breathing ceases for a short time. The highest temperature has been 102, the lowest 99.6. He speaks rationally and complains of pain in the abdomen.

A case of intrathoracic tumour was also shown, occurring in a man thirty-one years of age, who had been a soldier. He presented a slight, very tender, pulsating tumour about the second right intercostal cartilage. No bruit could be detected over this, and the heart sounds were muffled. It was believed to be a case of aortic aneurysm.

After examining the cases in the wards, the members of the society met together in Dr. Lloyd's private room for the purpose of discussion, the president, Dr. Savill, being in the chair. With regard to the case of blenorrhagic arthritis, Dr. Harris asked if his helpless condition might not be due to a condition of the vertebræ. Dr. Hopkins pointed out that the nails were altered in shape, and there was a rash on the skin. He would like to suggest that possibly the joint lesion had given rise to a nerve lesion, and this to the skin eruption. Dr. Savill said that if it was a case of blenorrhagic arthritis it was one of general arthritis. This differed from the ordinary description which was given in books, which description he believed to be incorrect. With regard to the case of obesity, Dr. Lloyd remarked that although the woman was 70 years of age, she had not suffered from any kidney mischief. There never was albumen, and now there were no uric acid crystals in the urine, notwithstanding the strictly proteid diet. There was no history of alcoholism in her case. Dr. Cross had under his care a case of obesity, but being complicated with chronic ulcer of the leg, he did not like to give her a strictly meat diet. Dr. Webster remarked it was curious that with such a strict proteid diet there were no crystals of uric acid in the urine. He suggested that this should be treated with dilute hydrochloric acid, and the deposit thus obtained weighed and compared with the normal standard. Dr. Hopkins referred to the Masai, who, when they went to war, killed an ox and ate an enormous quantity of meat, and then abstained from flesh for a month. Dr. Lloyd stated that the French doctors directed their patients to abstain from bread and pastry for treatment of this complaint.

Concerning the case of probable aneurysm, Dr. Blachford said one could not exclude the possibility of its being a case of neoplasm. Dr. Cross said that there was a considerable amount of pain present for such a small aneurysm; he should suggest treating it with iodide of potassium. Dr. Harris believed that tenderness was common, and especially so when clotting of the blood was taking place in the aneurysmal sac. Concerning the patient with total loss of memory, Dr. Savill considered it a most interesting one. A case of a similar character was first described by Charcot. Dr. Hopkins remembered a somewhat similar case occurring in a man, who, in writing, would keep repeating the same letters as did the patient under discussion. In discussing the case of skin disease Dr. Webster thought considering its tubercular aspect it might be benefited by the use of an ointment of salicylic acid. Dr. Savill suggested that from its appearance and position, together with the fact that mercury and iodide had failed to benefit it, it might be a case of senile scrofula. Dr. Cross thought the dose of the iodide ought to be increased. Dr. Blachford, in referring to the case of tetanus in a child, stated that tetanus was rare without a skin lesion, and although he had carefully examined the patient all over, he had failed to detect one in this case. Apparently there were no worms present in the intestinal tract. He suggested the possibility of its being brought on by pediculi capitis, the boy's head being full of lice on admission. He was losing the spasms, but was becoming weaker. Dr. Cross said that four years ago he had under his care four cases of tetanus occurring in adults. At first sight they all appeared to be idiopathic in nature, but, on careful examination, he was able to discover local injuries in three. The case which appeared to be idiopathic got well, but the others died. The cause of death appeared to be high temperature. In one case he had allowed cold air to blow on a patient with high fever, with the result that the temperature fell eight degrees. But the patient eventually died, and the temperature in the rectum after death registered 109 degrees. In one case which died from spasm there was found to be a rupture of the rectus abdominis muscle. In all the cases, although the spinal cords had been examined microscopically, no lesion could be detected. Dr. Savill said the present view was that tetanus was due to a bacillus. He had had under his care a patient with tetanus coming on after a wound in the hand, who recovered, he believed, owing to the administration of chloral. He had taken as much as 120 grains in twenty-four hours. Vomiting ensued, the chloral was stopped, the spasms became very severe, and chloroform was administered. Chloral was then given by the rectum, the patient improved; after omission the spasms again occurred. Dr. Webster considered it most unusual to get a case of tetanus without a wound. Dr. Savill said idiopathic tetanus was stated to be common in India, and Dr. Harris referred to the case of an idiopathic origin in an undergraduate of the University, and who made a good recovery.

